# Supplementary material for: Aptamer-Based Detection of Ampicillin in Urine Samples
Source: Antibiotics (Basel). 2020 Sep 29;9(10):655. doi: 10.3390/antibiotics9100655 (PMC7601551; doi:10.3390/antibiotics9100655)
Supplement: Supplementary file 1 [file antibiotics-09-00655-s001.pdf]

antibiotics

Supplementary Materials
Aptamer Based Detection of Ampicillin in Urine Samples

Matthew Simmons [1]*, Lisa Miller [1,2]*, Ms Malin O. Sundström[1] and Steven Johnson[1,]*

[1] Department of Electronic Engineering, University of York, Heslington, York, North Yorkshire YO10 5DD,, , United Kingdom; matthew.simmons@york.ac.uk; steven.johnson@york.ac.uk
[2] Department of Chemistry, University of York, Heslington, York, North Yorkshire, YO10 5DD, United Kingdom; lisa.miller@york.ac.uk

* Correspondence: matthew.simmons@york.ac.uk, steven.johnson@york.ac.uk; Tel.: +44 (0)1904 322693 (S.J.)

Blank Control Assay – No labelled Aptamer Present

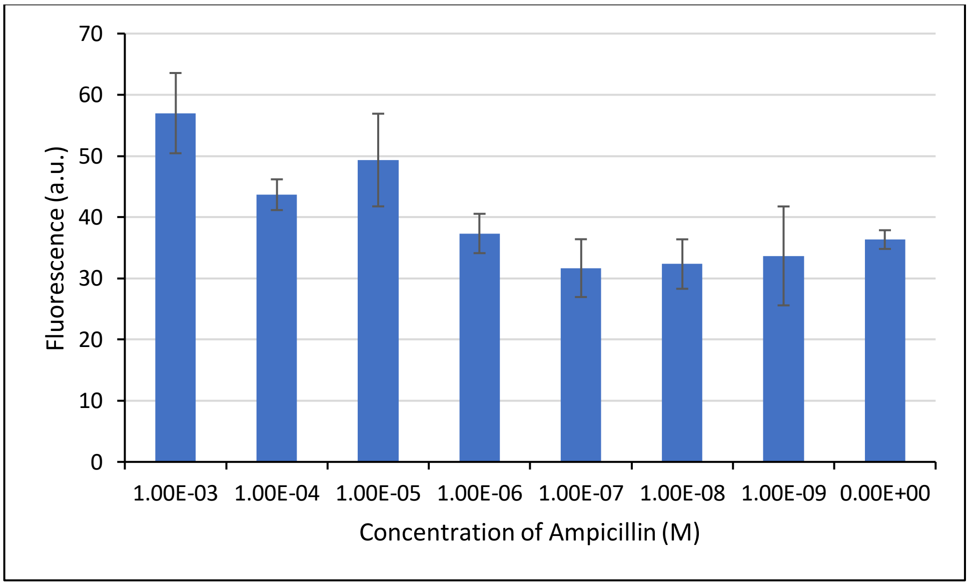

Figure S1. Blank control assay, performed at FAM fluorescence frequencies (λ_{ex} = 487 nm, λ_{em} = 528 nm) when no labelled aptamer is present and exposed to varying concentrations of ampicillin in buffer. The fluorescence observed is negligible in comparison to that of the fluorescence when the aptamer is present, and no correlation can be seen between the concentration of ampicillin and the fluorescence.

Control Assay – Using Non-Selective Labelled Aptamer Strand (FAM (3′-TATGCGCCGGTTTTCAGCCT-5′-FAM))

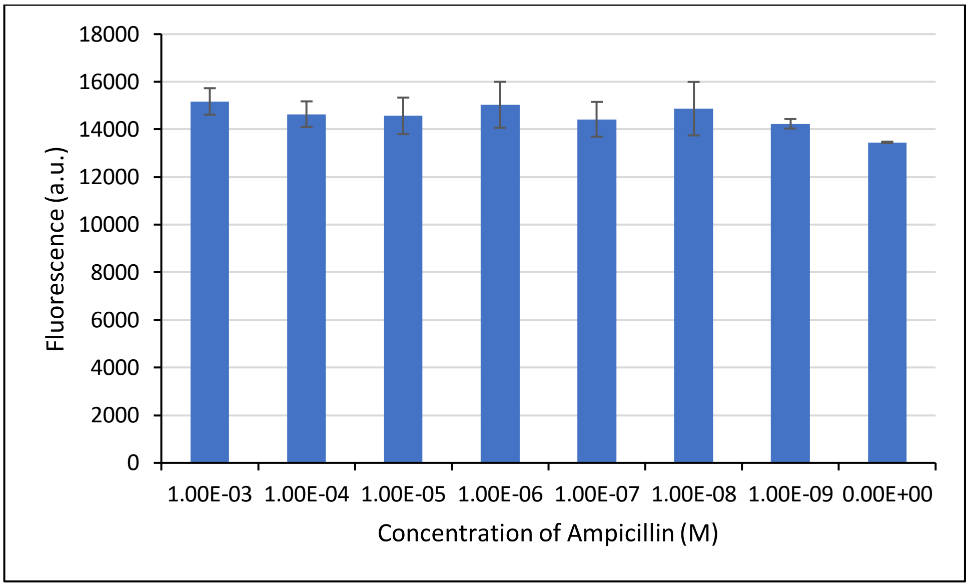

Figure S2. Control Data for the assay when using a FAM labelled control aptamer (not specific to ampicillin) in KPi buffer in the presence of varying concentrations of ampicillin. There can be seen to be no correlation between the degree of fluorescence of the assay and the concentrations of ampicillin.

^{1}H NMR and Mass Spectrometry Data for Ampicillin

^{1}H NMR (400 MHz, DMSO-d$_6$) δ 7.42 (d, *J* = 7.1 Hz, 2H), 7.33 (dt, *J* = 15.0, 7.2 Hz, 3H), 5.48 (d, *J* = 4.0 Hz, 1H), 5.39 (d, *J* = 4.0 Hz, 1H), 4.69 (s, 1H), 4.10 (s, 1H), 1.54 (s, 3H), 1.41 (s, 3H).

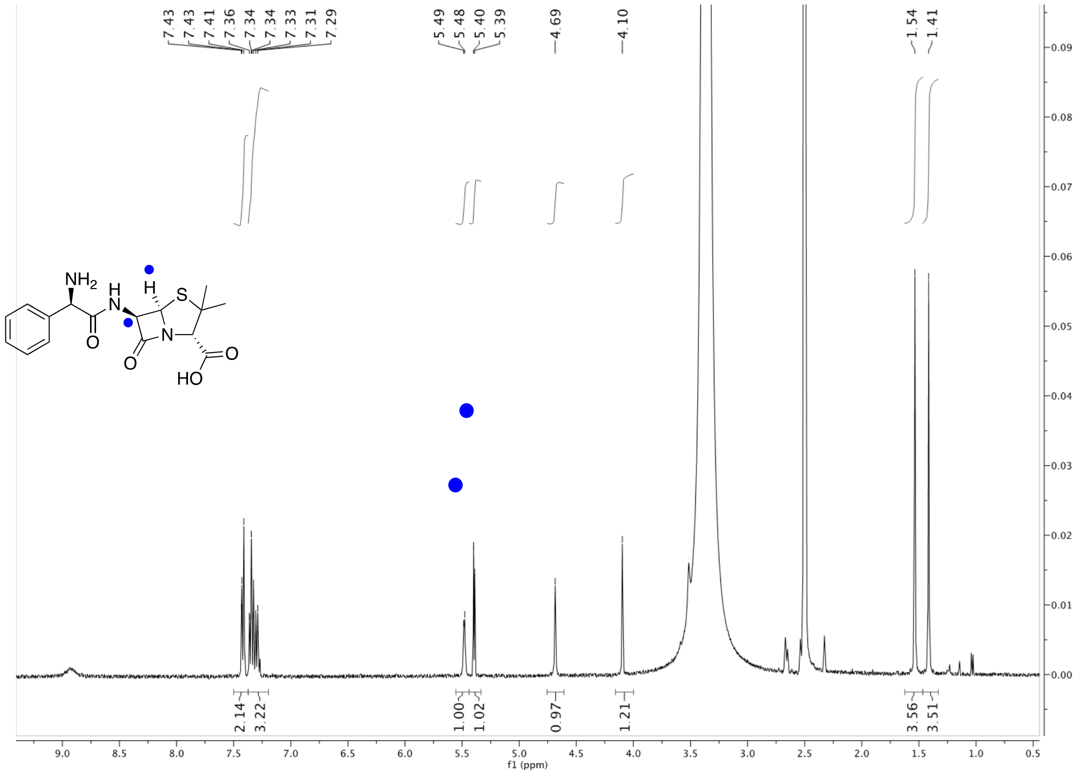

Figure S3. ^{1}H NMR spectrum of Ampicillin in DMSO-d$_6$ with β-lactam protons labelled.

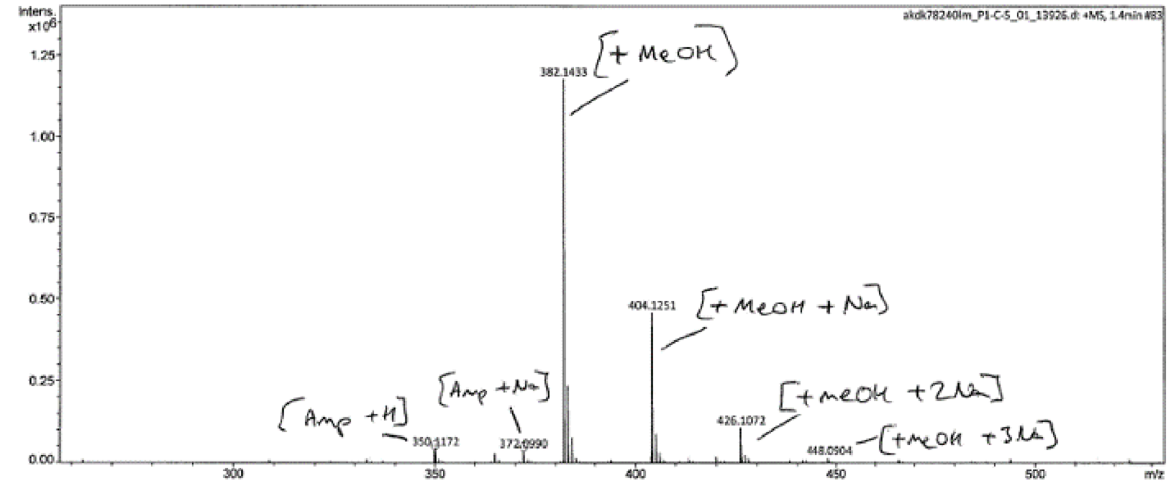

Meas. m/z	#	Ion Formula	m/z	err [ppm]	err [mDa]	mSigma	Mean err [ppm]
350.1172	1	C16H20N3O4S	350.1169	-0.9	-0.3	9.7	-0.3
372.0990	1	C16H19N3NaO4S	372.0988	-0.4	-0.2	18.0	-1.1

Figure S4. Mass spectrometry analysis of Ampicillin in MeOH

^{1}H NMR and Mass Spectrometry Data for Hydrolysed Ampicillin

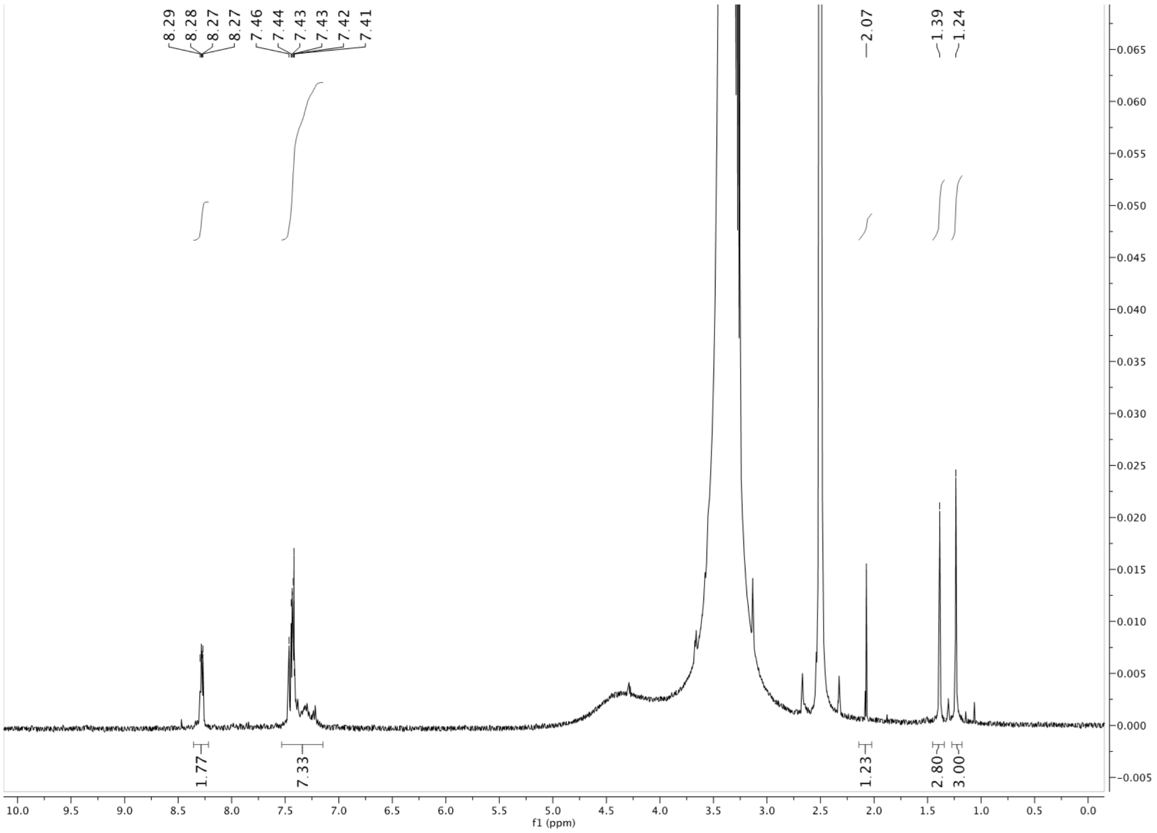

Figure S5. ^{1}H NMR spectrum of Hydrolysed Ampicillin in DMSO-d_6

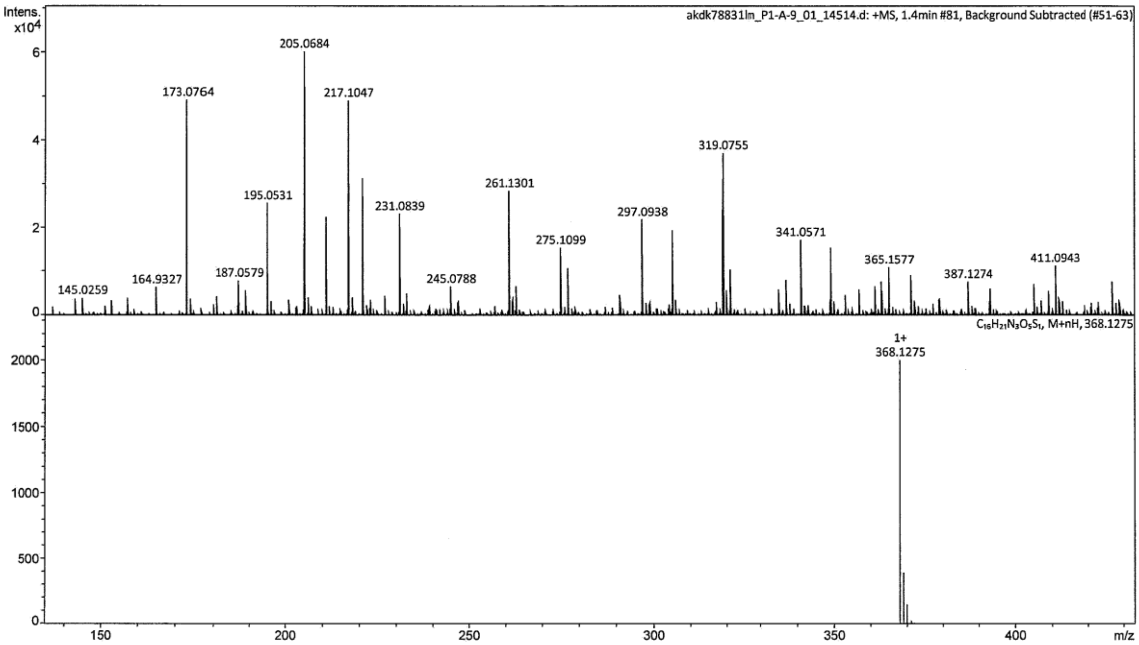

Figure S6. Mass Spectormetry data for Hydrolysed Ampicillin in MeOH

2D Fluorescence Spectra of Alexafluor647 and FAM labelled aptamer, Urine, Ampicillin and Hydrolysed Ampicillin

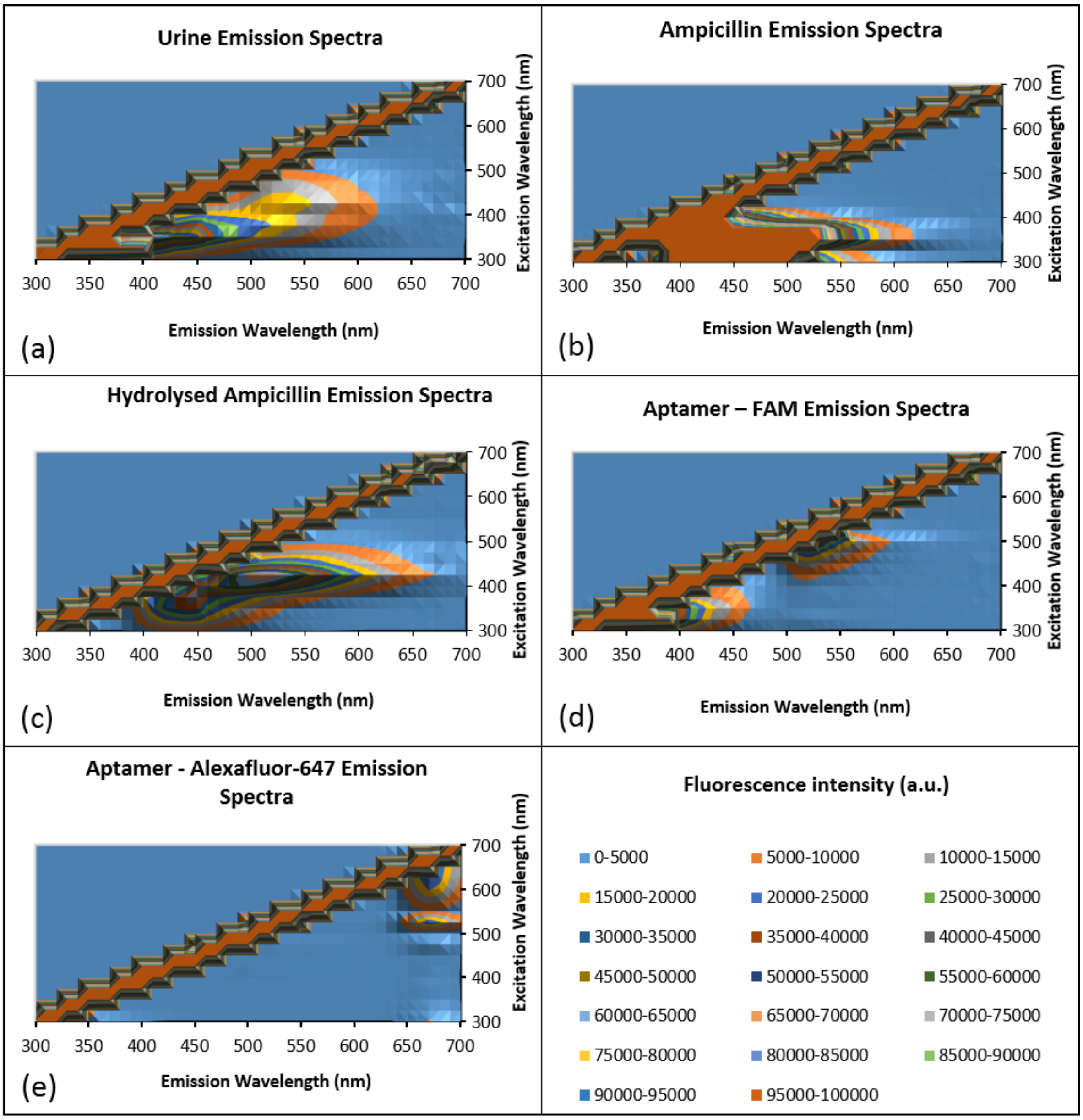

Figure S7. 2D fluorescence emission/excitation spectra of the main assay components. The broad fluorescence of the Urine (**a**), ampicillin (**b**), and hydrolysed ampicillin (**c**) clearly overlap and interefere with that of the FAM labelled aptamer strand (**d**). The Alexafluor647 labelled aptamers emission and excitation are clearly unaffected by these.

Blank Control Data for the Final Assay in both Urine and Buffer

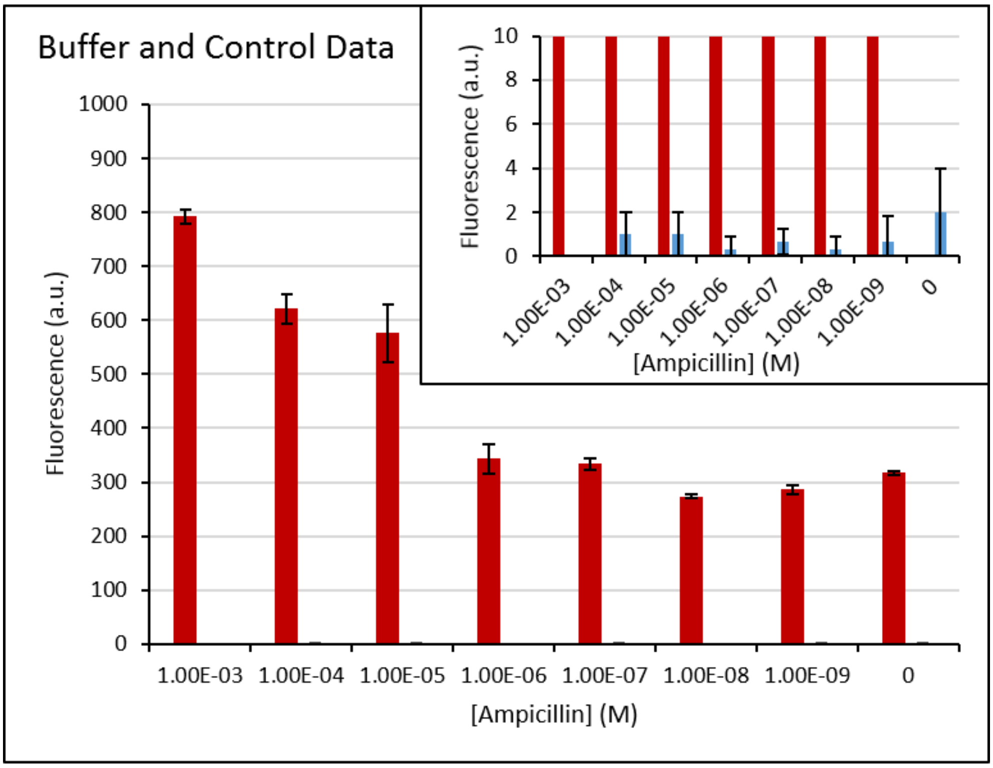

Figure S8. Blank Control data for final assay using the Alexafluor647 labelled aptamer (red) in buffer in the presence of varying concentrations of ampicillin. Inset magnification to show levels of fluorescence present when no Alexafluor647 labelled aptamer is present (blue). The levels of fluorescence can be seen to negligible in comparison to that of the assay in the presence of ampicillin.

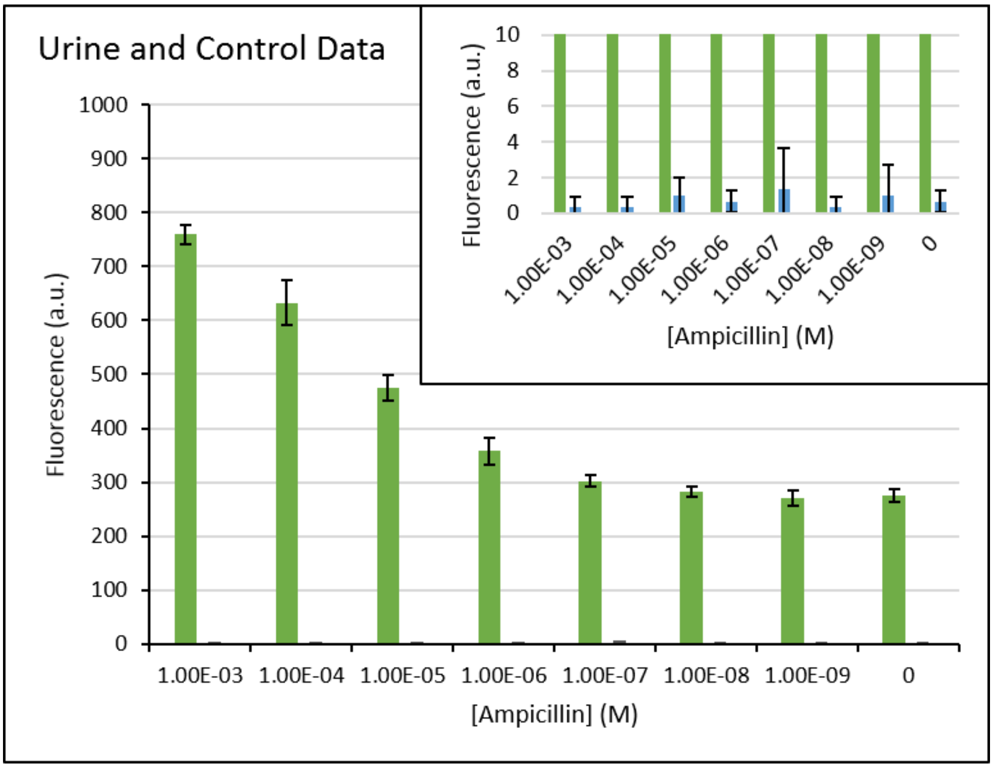

Figure S9. Control data for final assay using the Alexafluor647 labelled aptamer (green) in urine in the presence of varying concentrations of ampicillin. Inset magnification to show levels of

fluorescence present when no Alexafluor647 labelled aptamer is present (blue). The levels of fluorescence can be seen to negligible in comparison to that of the assay in the presence of ampicillin.
